# Supplementary material for: Development of an automatic segmentation system for anterolateral thigh flap perforators in maxillofacial reconstruction
Source: PLoS One. 2026 Feb 23;21(2):e0342109. doi: 10.1371/journal.pone.0342109 (PMC12928459; doi:10.1371/journal.pone.0342109)
Supplement: Attachment — Submitted filename: PlosOne_Response to reviewers.docx [file pone.0342109.s002.docx]

| **Date:** | Mar 17 2025 02:13PM |
| --- | --- |
| **To:** | "In-Seok Song" densis@korea.ac.kr |
| **From:** | "PLOS ONE" plosone@plos.org |
| **Subject:** | PLOS ONE Decision: Revision required [PONE-D-24-59305] |

PONE-D-24-59305

Development of an automatic segmentation system for anterolateral thigh flap perforators in maxillofacial reconstruction

PLOS ONE

Dear Dr. Song,

Thank you for submitting your manuscript to PLOS ONE. After careful consideration, we feel that it has merit but does not fully meet PLOS ONE’s publication criteria as it currently stands. Therefore, we invite you to submit a revised version of the manuscript that addresses the points raised during the review process.

Please submit your revised manuscript by May 01 2025 11:59PM. If you will need more time than this to complete your revisions, please reply to this message or contact the journal office at [plosone@plos.org](mailto:plosone@plos.org). When you're ready to submit your revision, log on to https://www.editorialmanager.com/pone/ and select the 'Submissions Needing Revision' folder to locate your manuscript file.

Please include the following items when submitting your revised manuscript:

- A rebuttal letter that responds to each point raised by the academic editor and reviewer(s). You should upload this letter as a separate file labeled 'Response to Reviewers'.
- A marked-up copy of your manuscript that highlights changes made to the original version. You should upload this as a separate file labeled 'Revised Manuscript with Track Changes'.
- An unmarked version of your revised paper without tracked changes. You should upload this as a separate file labeled 'Manuscript'.

If you would like to make changes to your financial disclosure, please include your updated statement in your cover letter. Guidelines for resubmitting your figure files are available below the reviewer comments at the end of this letter.

If applicable, we recommend that you deposit your laboratory protocols in protocols.io to enhance the reproducibility of your results. Protocols.io assigns your protocol its own identifier (DOI) so that it can be cited independently in the future. For instructions see: <https://journals.plos.org/plosone/s/submission-guidelines#loc-laboratory-protocols>. Additionally, PLOS ONE offers an option for publishing peer-reviewed Lab Protocol articles, which describe protocols hosted on protocols.io. Read more information on sharing protocols at <https://plos.org/protocols?utm_medium=editorial-email&utm_source=authorletters&utm_campaign=protocols>.

We look forward to receiving your revised manuscript.

Kind regards,

Shimpei Miyamoto
Academic Editor
PLOS ONE

**Reviewer #1:** I have several points for this paper to be finalized.
Abstract
1. The purpose of this study in abstract is written twice.

→ Thank you for your kindness comments. We removed redundancy and revised the purpose of the study to one sentence as follows:

Page 2, Line 7

“To address this challenge, we developed and validated a CNN-based automatic segmentation model for detecting ALT flap perforators on comuted tomography angiography (CTA).”

M&M
2. Study subjects and data collection - all reviewed patient's information should be presented in this section (gender, age, BMI)

→ Thank you for your kindness comments.

Page 4, Line 74

“The mean age was 62.1 years (range, 35–84 years), and the mean BMI was 23.5 kg/m² (range, 18.99–34.85 kg/m²).”

3. training, validation and test set number is described in very confused way. In total, manual segmentation for 69 patients (138 image sets) were done?

→ We are apologizing for the confusion. We have described the dataset split in more detail as follows.

Page 5, Line 87

“A total of 69 patients were included for analysis. Among these, 56 patients were assigned …”

4. The term "ground truth and manual mapping" is used in confusion way. For train as well as test sets, ground truth is just ground truth. Also, the term mapping cannot be used as the replacement of segmentation.

→ Thank you for your helpful comment. We agree with your suggestion and have revised the terminology throughout the manuscript. "Ground truth" is consistently used to refer to the manually annotated segmentation used for both training and evaluation. In addition, we have replaced the term "mapping" with "segmentation" to avoid confusion.

5. The manual segmentation method should be described with more detail. Did clinician draw boundary of the vessel to determine ROI? Or identified center and used seed grown method? This is also related to the clinical evaluation.

→ Thank you for your insightful comment. The ROI was manually designated in the coronal plane, extending from the iliac crest to the superior margin of the patella. Within this predefined region, a clinician manually identified and annotated the artery location on each image slice based on visual assessment. This process did not rely on automated techniques such as seed-growing or centerline tracking, but was instead performed entirely through expert manual delineation.

Page 4, Line 80

“The region of interest (ROI) was manually defined in the coronal plane ...”

6. In clinical evaluation, the difference between the center of ROI was obtained? If it is so, the determining method of the center of ROI should be stated. It is not clearly written.

→ Thank you for the valuable comment. We would like to clarify that no data were collected regarding the difference between the centers of the ROIs. Therefore, the method for determining the center of the ROI was not applicable in this study.

7. It is difficult to understand how the method implemented in the clinical evaluation can be considered a clinical evaluation. It would be better to refer to the previous literature further. This evaluation method dose not fully understand as the way that clinician used in actual clinical conditon.

→ Thank you for your insightful comment. The clinical relevance of this study lies in the fact that accurate preoperative identification of skin perforators in the anterolateral thigh is essential for surgical planning, particularly in reconstructive procedures. AI-driven perforator localization provides valuable information that can aid clinicians in flap design and reduce intraoperative uncertainty. In this study, clinical evaluation was conducted by comparing the AI-predicted perforator locations with those manually traced by experienced clinicians, which reflects a feasible reference standard in the absence of intraoperative validation. We have also revised the manuscript to clarify the clinical context and added a relevant literature citation to support this evaluation approach.

Page 11, Line 247

“Although intraoperative validation was not performed in this study…”

Reference

36. Garvey, Patrick B., et al. "A prospective study of preoperative computed tomographic angiography for head and neck reconstruction with anterolateral thigh flaps." Plastic and reconstructive surgery 127.4 (2011): 1505-1514.

Result
Overall result is very confusing because it shows too much information. Some are not related to the study purpose and some are not necessary to support study hypothesis.

8. Subject distribution - I would suggest to present a table for patient's general information. Also, is it necessary to to describe the type of perforator according to sex?

→ Thank you for your valuable suggestion. As also addressed in our response to Comment 2, we have included a summary of patient demographics, including gender, age, and BMI, in the revised manuscript (Page 4, Line 74).

Regarding the suggestion to classify perforator types by sex, we respectfully consider that classifying perforator types by sex is not clinically meaningful in the context of anterolateral thigh flap planning. While anatomical variations may exist between individuals, perforator characteristics are primarily influenced by patient-specific vascular anatomy rather than sex. Therefore, sex-based subgroup analysis was not deemed necessary in this study.

9. There must be explanation for the difference between the right and left side.

→ Thank you for this important observation. As suggested, we have added an interpretation in the Discussion section regarding the statistically significant differences observed between the left and right thighs in Table 3.

Page 11, Line 254

“Interestingly, segmentation performance showed a statistically significant difference between the left and right thighs…”

10. Some results are not suitable for the study purpose, for example figure 2. This study is not for showing distribution of vessel position.

→ Thank you for your valuable comment. Although no statistical analysis was conducted due to the limited sample size, Figure 2 was initially included to visually convey that perforator tended to cluster in the distosuperior quadrant. We believed this visual trend could help readers intuitively understand the spatial distribution observed in our dataset. However, we agree that this figure is not directly aligned with the core objective of the study. To maintain focus and clarity, we have removed Figure 2 from the revised manuscript.

Discussion
11. the first paragraph should be in introduction section.

→ Thank you for your kindness comment. As suggested, we have moved the first paragraph of the Discussion section, which provides contextual background on the clinical relevance of ALT flap perforator detection, to the Introduction section.

12. The third paragraph is not very important according to the study purpose, except the last sentence. Although, the last sentence seems broken and don't understand. (This study's most challenging detections involved perforators traveling through muscles before reaching subcutaneous layers.)

→ Thank you for your valuable feedback. We included the third paragraph to help readers understand the clinical implications of different perforator types, as we believe this information provides important context for interpreting the relevance and limitations of the proposed method. We agree, however, that the final sentence of the paragraph lacked clarity. Accordingly, we have revised the sentence for improved readability as follows: The most challenging cases involved musculocutaneous perforators that traveled through the muscle before reaching the subcutaneous layer, which may affect the model’s detection accuracy.

Page 9, Line 197

“Nevertheless, the most challenging cases often involved musculocutaneous perforators …”

13.In 4th paragraph, "the progression course of the artery could be confirmed more clearly in younger women with sufficient subcutaneous tissue thickness in.." -> This part was not stated either in method nor result. It cannot be discussed as the readers do not have any idea about it.

→ Thank you for your helpful comment. The statement regarding clearer confirmation of the arterial course in younger women with sufficient subcutaneous tissue was based on subjective impressions observed during the manual mapping process. However, as this observation was not supported by objective data and was not included in the Methods or Results sections, we agree that it is inappropriate to include it in the Discussion. Accordingly, we have removed this sentence from the revised manuscript.

14.In 4th paragraph, "On the other hand, there was no statistical difference in accuracy depending on the type..." This part needs to be discussed about.

→ Thank you for your thoughtful comment. In response, we have expanded the discussion to provide interpretation of the non-significant differences in segmentation accuracy according to perforator type, sex, and BMI. These findings may be explained by the fact that our deep learning model focused on structural features in the arterial region regardless of perforator classification or body composition. In addition, the relatively small sample size and the controlled imaging conditions may have minimized the potential variation across subgroups.

Page 10, Line 206

“On the other hand, there was no statistical difference in accuracy depending on the type of …”

15. 5th paragraph can be removed or reduced.

→ Thank you for the comment. We agree that the fifth paragraph contained general information that may not be essential for the discussion. Accordingly, we have removed the introductory descriptions of image classification, segmentation, and detection, and retained only a brief explanation to clarify why segmentation was used in the present study.

Page 10, Line 213

“In this study, segmentation was adopted to localize the perforator boundaries precisely …”

16. 7th paraphragh, "The main trunk starting from the femoral artery near the hip joint had a large diameter...." I cannot find any description about this information, it suddenly appeared in disscusion only. I would suggest to present result of DSC, JSC if there is significancy according to the superior1/3, middle1/3, inferior1/3 part. If it is presented, the above sentence can be supported.

→ Thank you for your valuable comment. We agree that the segmentation accuracy (DSC, JSC) is likely influenced by the vessel diameter, particularly along the course of the perforator from the main trunk to the distal branches. While we considered performing a subgroup analysis based on the anatomical thirds (superior, middle, inferior) to reflect these diameter-related differences, the distinction between the main trunk, mid-branch, and perforator is often not clearly demarcated in practice, especially in CTA images. Although the main trunk can be identified relatively easily, the transition to smaller branches, particularly the musculocutaneous or septocutaneous perforators, lacks consistent anatomical boundaries, making objective classification for analysis difficult. Although we did not perform a separate quantitative analysis by vessel diameter, we believe that the lower DSC and JSC values observed near the distal ends of the perforators are plausibly related to smaller diameters and more complex branching patterns, given the anatomical and imaging characteristics of these regions. We have added a note in the Discussion section to address this, and we plan to explore a more detailed stratification of segmentation performance according to vessel diameter or anatomical subregion in a future study.

Page 12, Line 269

“Additionally, segmentation performance tended to decrease toward the distal ends of the perforators …”

17. 8th paragraph is not very related to the study purpose or result?

→ Thank you for your comment. While we agree that the eighth paragraph does not present new quantitative results, we included this section to provide additional clinical interpretation and contextual relevance by referencing prior anatomical literature. Specifically, the A–P line and ABC system were discussed to support the validity of our detection results and to highlight the potential for future integration into clinically standardized flap design protocols. We have revised the paragraph slightly to clarify its purpose and relevance to the study.

18. 9th paraphraph is the repeatition of the previous paragraphs, needs to be revised.

→ Thank you for your comment. We agree that the 9th paragraph included content that overlapped with earlier sections. Accordingly, we have revised this paragraph to remove redundancy and retain only the key message regarding the clinical implications of the distance differences between manual and automatic detection. Additional discussion on future research directions has been added to enhance the relevance of this section.

Page 11, Line 241

“For clinical evaluation, the distance between the manually and automatically detected …”

19. 10th paragraph, "First, the n value to proceed": "n value" -> sample size

→ Sorry for the confusion. We have revised the phrase "n value" to "sample size" to improve clarity and adhere to appropriate academic terminology in the revised manuscript.

**Reviewer #2:** Authors of this study propose an innovative methodological solution to facilitate the identification of the vessels of the ALT pedicle. The study focuses on analysis and modeling, by a neural network of the path, of the perforating artery, and its comparison with manual modeling by an experimented operator. The objective of the study seems to be the feasibility of modeling the path of pedicle by AI software. The comparison with manual modeling serves as extrinsic validation. The primary endpoint is the accuracy of the location of the perforator on imaging. The means of evaluation is the comparison of projection concordance tests on imaging. The secondary endpoints seem to be the influence of gender, side, BMI and muscular or septal location of the perforators.
The study has a small number of patients: 56 patients for machine learning (plus 11 excluded), 13 patients for the control after learning. Both legs were analyzed for each patient. The software performed the detection on the 2D scanner, then reconstructed in 3D the path of the vessel, and the projection of the perforator to the skin. Changes in size, contrast, and angulation of the images were applied randomly to eliminate algorithmic bias. Concordance tests for the detection of the perforator on the slices were performed.
The results found a statistically significant projection concordance for the left side, not significant for the right side. The discussion clarifies some aspects and puts this study into perspective with the existing literature. In addition, they take into consideration the limitations of this study, particularly in terms of patient numbers.
This paper is interesting, providing a relevant solution to simplify the identification of perforators, however, it seems to me that the work is not entirely complete.

1. The objective of the study seems a little imprecise. Is the objective to demonstrate the feasibility of automatic segmentation, or to show that automatic segmentation performs as well as manual segmentation?

→ Thank you for this important point. We clarified the study objective in both the abstract and conclusion. The primary aim of this study was to evaluate the feasibility and accuracy of an automatic segmentation model for detecting ALT flap perforators using CTA. In doing so, we compared the performance of the automatic method against expert manual annotations to validate its clinical applicability.

Page 2, Line 7

“To address this challenge, we developed and validated a CNN-based automatic segmentation …”

Page 12, Line 270

“In conclusion, this study demonstrates the feasibility of deep learning …”

2. The primary judgment criterion could be specified: is it a comparison on 2D slices or on 3D reconstructions?

→ Thank you for your thoughtful comment. While segmentation was performed on individual axial 2D slices, we clarified that all quantitative accuracy metrics (DSC, JSC) were computed on a per-patient basis by averaging the scores across each patient's slices.

Page 7, Line 130

“All accuracy metrics were calculated per patient by averaging across the segmented slices of each case. Although the segmentation was performed at the slice level, the final evaluation was based on patient-level aggregation to reflect clinical relevance.”

3. It seems relevant to me to formalize the expression of the secondary judgment criteria, these do not seem to be perfectly defined in the article.The comparison of projection concordance tests seems to be adapted to the demonstration of the objective of the study and perfectly conducted. The statistical tests seem relevant to me.

→ Thank you for your valuable comment. In the revised manuscript, we have clearly defined the secondary judgment criteria in a newly added subsection titled “Evaluation Criteria” within the Methods section.

Page 6, Line 118

4. In the results and the discussion, I did not understand whether the computer analysis allowed to detect the septal or muscular path of the perforators, or whether the margin of error did not allow to conclude.

→ Thank you for your insightful comment. The deep learning model used in this study was designed to perform automatic segmentation of the perforator region but was not trained to classify the perforator progression type (i.e., septocutaneous vs. musculocutaneous). The classification of perforator type was performed manually based on anatomical evaluation of the CTA images. While the model successfully segmented both types, the distinction between septal and muscular paths was not part of the model’s output.

5. The conclusion of the study could be specified. We can conclude that automatic segmentation is possible, but, within the limits of this experimental procedure, seems to still lack a little precision.

→ Thank you for this important comment. We have revised the conclusion to clearly reflect that the proposed automatic segmentation system demonstrates the feasibility of perforator detection, while also acknowledging the limitations of the current experimental setting. Specifically, we noted that the model’s spatial precision, particularly in localizing terminal points, still requires improvement. We also suggested future work involving larger datasets and refined model training to enhance both accuracy and clinical reliability.

Page 12, Line 270

“In conclusion, this study demonstrates the feasibility of deep learning …”

**Reviewer #3:** This study addresses the challenge of identifying perforators in the anterolateral thigh (ALT) flap for maxillofacial reconstruction by developing an automatic segmentation system, directly serving clinical practice and aligning with the practical needs of plastic surgery. Additionally, it pioneers the application of deep learning in the automatic segmentation of ALT flap perforators, filling a gap in this field and demonstrating significant innovation. However, some issues may need to be addressed.

1. The small sample size could limit the generalizability of the model, especially in clinical settings where diverse anatomical variations exist. Although the authors use manual mapping as the ground truth for training the model, relying on a single researcher for this step introduces a potential source of bias.

→ Thank you for pointing this out. We acknowledge that the limited sample size may constrain the generalizability of our findings, particularly considering the anatomical diversity observed in clinical practice. To address this limitation, we have explicitly mentioned it in the revised Discussion section and have added a statement in the Conclusion to highlight the need for larger, multi-institutional datasets in future studies. Additionally, we agree that reliance on a single annotator may introduce bias; however, to ensure consistency and reduce inter-observer variability during model training, annotations were performed by a clinician with substantial experience in ALT flap mapping. In future work, we plan to incorporate multi-expert validation and inter-rater reliability assessment to strengthen annotation robustness.

2. The reported distance differences between manual and automatic detection of perforators (e.g., 38.28 ± 15.52 mm on the left side) suggest some degree of inaccuracy. This could potentially be problematic for clinical applications where precise identification of perforators is crucial for successful surgical planning.

→ Thank you for this important observation. We agree that precise localization is essential for surgical planning. Although the reported distance differences indicate some spatial deviation, we believe the automatically detected perforator regions still provide clinically meaningful guidance. In practice, perforator selection is influenced not only by absolute point localization but also by regional vascular patterns, the proximity of reliable vessels, and the overall anatomical landscape. Our model consistently localized perforators within the expected anatomical zone and demonstrated high segmentation overlap (DSC/JSC), which may be sufficient for preoperative planning, especially in cases requiring rapid or preliminary evaluation. We have clarified this point in the revised Discussion and emphasized that, while further refinement is needed, the current system offers clinically valuable assistance in flap design.

3. The references are outdated and may not reflect the latest advancements in imaging technologies and the application of deep learning in medical practices.

→ Thank you for your helpful comment. We reviewed and updated the references to include more recent publications (within the past 5 years) that reflect current advancements in both deep learning applications in medical image segmentation and perforator flap planning.

6. PLOS authors have the option to publish the peer review history of their article ([what does this mean?](https://journals.plos.org/plosone/s/editorial-and-peer-review-process#loc-peer-review-history)). If published, this will include your full peer review and any attached files.


If you choose “no”, your identity will remain anonymous but your review may still be made public.


**Do you want your identity to be public for this peer review?** For information about this choice, including consent withdrawal, please see our [Privacy Policy](https://www.plos.org/privacy-policy).

Reviewer #1: No

Reviewer #2: **Yes:** Franck Delanoë

Reviewer #3: No

[NOTE: If reviewer comments were submitted as an attachment file, they will be attached to this email and accessible via the submission site. Please log into your account, locate the manuscript record, and check for the action link "View Attachments". If this link does not appear, there are no attachment files.]

While revising your submission, please upload your figure files to the Preflight Analysis and Conversion Engine (PACE) digital diagnostic tool, <https://pacev2.apexcovantage.com/>. PACE helps ensure that figures meet PLOS requirements. To use PACE, you must first register as a user. Registration is free. Then, login and navigate to the UPLOAD tab, where you will find detailed instructions on how to use the tool. If you encounter any issues or have any questions when using PACE, please email PLOS at [figures@plos.org](mailto:figures@plos.org). Please note that Supporting Information files do not need this step.

*In compliance with data protection regulations, you may request that we remove your personal registration details at any time.* [*(Remove my information/details)*](https://www.editorialmanager.com/pone/login.asp?a=r)*. Please contact the publication office if you have any questions.*
