# Supplementary material for: Development of an automatic segmentation system for anterolateral thigh flap perforators in maxillofacial reconstruction
Source: PLoS One. 2026 Feb 23;21(2):e0342109. doi: 10.1371/journal.pone.0342109 (PMC12928459; doi:10.1371/journal.pone.0342109)
Supplement: Attachment — Submitted filename: PlosOne_Response to reviewers_251129.docx [file pone.0342109.s003.docx]

Revision

PONE-D-24-59305R1

Development of an automatic segmentation system for anterolateral thigh flap perforators in maxillofacial reconstruction

PLOS ONE

Dear Dr. Song,

Thank you for submitting your manuscript to PLOS ONE. After careful consideration, we feel that it has merit but does not fully meet PLOS ONE’s publication criteria as it currently stands. Therefore, we invite you to submit a revised version of the manuscript that addresses the points raised during the review process.

Please submit your revised manuscript by Nov 27 2025 11:59PM. If you will need more time than this to complete your revisions, please reply to this message or contact the journal office at [plosone@plos.org](mailto:plosone@plos.org). When you're ready to submit your revision, log on to <https://www.editorialmanager.com/pone/> and select the 'Submissions Needing Revision' folder to locate your manuscript file.

Please include the following items when submitting your revised manuscript:

- A rebuttal letter that responds to each point raised by the academic editor and reviewer(s). You should upload this letter as a separate file labeled 'Response to Reviewers'.
- A marked-up copy of your manuscript that highlights changes made to the original version. You should upload this as a separate file labeled 'Revised Manuscript with Track Changes'.
- An unmarked version of your revised paper without tracked changes. You should upload this as a separate file labeled 'Manuscript'.

If you would like to make changes to your financial disclosure, please include your updated statement in your cover letter. Guidelines for resubmitting your figure files are available below the reviewer comments at the end of this letter.

If applicable, we recommend that you deposit your laboratory protocols in [protocols.io](http://protocols.io/) to enhance the reproducibility of your results. Protocols.io assigns your protocol its own identifier (DOI) so that it can be cited independently in the future. For instructions see: [https://journals.plos.org/plosone/s/submission-guidelines#loc-laboratory-protocols](https://track.editorialmanager.com/CL0/https:%2F%2Fjournals.plos.org%2Fplosone%2Fs%2Fsubmission-guidelines%23loc-laboratory-protocols/1/010f0199dda87968-c5218c81-b482-4bb0-a0af-f290946724d0-000000/U9XkoyT7D-BCWQMVPyDkAb4LtSRIWD43eyvTBgf54Qc=232). Additionally, PLOS ONE offers an option for publishing peer-reviewed Lab Protocol articles, which describe protocols hosted on [protocols.io](http://protocols.io/). Read more information on sharing protocols at [https://plos.org/protocols?utm_medium=editorial-email&utm_source=authorletters&utm_campaign=protocols](https://track.editorialmanager.com/CL0/https:%2F%2Fplos.org%2Fprotocols%3Futm_medium=editorial-email%26utm_source=authorletters%26utm_campaign=protocols/1/010f0199dda87968-c5218c81-b482-4bb0-a0af-f290946724d0-000000/9x5F9m14wboei5loTzOVUrw0sdDyZwleTNhTk8eY1Gs=232).

We look forward to receiving your revised manuscript.

Kind regards,

John Minh Le, MD, DDS
Academic Editor
PLOS ONE

Reviewer #2: Thank you for the corrections you have made; they make your manuscript much more relevant.

In essence, it does indeed seem necessary to increase the number of subjects in the machine learning process to improve accuracy. The margin of error is a little too large for clinical application, and the lack of differentiation between the septal and muscular pathways is regrettable.

As a supplementary conclusion to this work, it could be suggested that this methodology may be applied to other perforator flaps.

Thank you for this work.

>> We sincerely appreciate the reviewer’s constructive comments. As suggested, we have clarified the impact of dataset size on model performance by adding a statement in the Discussion emphasizing the need for future expansion through multi-center datasets. We also explicitly acknowledged the lack of perforator-type differentiation as a limitation and have added this point to the Limitations section.

*Discussion, Page 9,*

*Because the dataset size was relatively modest, expanding the cohort, particularly through multi-center integration, is expected to further improve model robustness and reduce performance variability.*

*Discussion, Page 12,*

*Although the manual annotations distinguished septocutaneous and musculocutaneous perforators, the current model did not incorporate this classification, which remains an area for future methodological extension.*

Reviewer #3: This is an excellent research. The author's response cleared up my doubts. I have no further comments!

>> We sincerely appreciate the reviewer’s positive feedback.

Reviewer #4: This manuscript presents a deep learning–based approach for automatic segmentation of anterolateral thigh (ALT) flap perforators on computed tomography angiography (CTA). The clinical relevance is clear: accurate perforator identification is critical for flap harvest in maxillofacial reconstruction, yet manual mapping is time-consuming and variable. The novelty lies in applying convolutional neural networks (CNNs) to this niche domain, using a cascaded 2D/3D segmentation strategy. Overall, the study is innovative, clinically relevant, and well-structured. However, several methodological and interpretive aspects merit clarification and strengthening.

My major Concerns are

1. Dataset size and annotation bias

The study uses 80 patients, which is relatively modest for training and validating deep learning models, especially given the high variability of ALT perforators.

Furthermore, all ground truth annotations were produced by a single researcher, which introduces potential systematic bias. Multi-annotator validation or inter-rater agreement data would strengthen the credibility of the reference standard.

>> We appreciate this important comment. To address concerns regarding annotation bias, we conducted an additional inter-rater validation. A second oral and maxillofacial surgery resident independently annotated 10 randomly selected cases. Agreement between the two annotators was quantified using the Dice similarity coefficient, demonstrating high consistency (mean DSC = 0.88 $\pm$ 0.04). We have incorporated this new information into the Methods and Discussion sections to strengthen the reliability of our ground truth annotations.

*Discussion, Page 9,*

*To assess annotation reliability, an additional inter-rater validation was performed. A second oral and maxillofacial surgery resident independently annotated 10 randomly selected cases, and agreement between the two annotators was quantified using the Dice similarity coefficient, demonstrating high consistency (mean DSC = 0.88 ± 0.04).*

2. Evaluation metrics and clinical relevance

The primary metrics (DSC, JSC) provide a measure of overlap but may not fully capture clinical usability, particularly in terms of perforator localization accuracy.

Reporting absolute localization error relative to skin landmarks (e.g., ASIS, patella) would make the findings more translatable to surgical practice.

>> We sincerely appreciate the reviewer’s valuable comment. Our clinical evaluation already measured the Euclidean distance between the manually and automatically detected terminal skin points of the perforator. We clarified this methodology and emphasized its relevance for clinical translation in the revised manuscript.

*Result, Page 8,*

*Although these values represent several centimeters of deviation, they reflect the inherent difficulty of localizing small-caliber perforators on CTA and still provide clinically meaningful guidance for preoperative flap planning.*

*Discussion, Page 12,*

*However, the localization error in this study was calculated solely as the Euclidean distance between terminal skin points without reference to anatomical surface landmarks such as the ASIS or patella. Incorporating landmark-normalized measurements in future work may enhance clinical applicability by providing a more precise correspondence to intraoperative surface anatomy.*

3. Perforator classification

The model segments perforators but does not distinguish between septocutaneous and musculocutaneous types, which is highly relevant for surgical planning.

Even if not part of the current model, the lack of this functionality should be explicitly acknowledged as a limitation.

>> We fully agree. Although manual annotations distinguished septocutaneous and musculocutaneous pathways, the current model did not incorporate this classification. We added a clear statement in the Limitations section indicating that perforator-type classification represents a clinically important future extension.

*Discussion, Page 13,*

*In addition, although the manual annotations distinguished septocutaneous and musculocutaneous perforators, the current model did not incorporate this classification. Integrating perforator-type differentiation into future models would enhance the clinical relevance of automated preoperative planning.*

4. Projection to surface anatomy

A critical challenge of CTA-based mapping is translating deep vascular anatomy to precise cutaneous points. The manuscript does not fully address this limitation.

A comparison with ultrasound-based localization, which directly identifies perforators at the skin surface, would contextualize the clinical implications more clearly.

>> Thank you for highlighting this point. We have revised the Discussion to emphasize that CTA identifies deep vascular anatomy but does not directly project perforator coordinates onto the skin surface, which limits intraoperative translation. Additionally, we have added a paragraph noting that ultrasound provides real-time surface-level localization, and future comparative work integrating CTA and ultrasound could further improve preoperative accuracy.

*Discussion, Page 12,*

*Moreover, ultrasound provides real-time identification of perforators at the skin surface, and future comparative work integrating CTA with ultrasound-based localization may further improve preoperative accuracy.*

Minor Comments

The Methods section would benefit from a more formal definition of secondary outcome measures and the rationale for statistical tests used.

>> We sincerely appreciate the reviewer’s helpful comment. We clarified secondary evaluation methods and added the rationale for statistical tests.

*Secondary Evaluation, Page 7,*

*The secondary outcome measure was the spatial localization error, defined as the Euclidean distance between manually and automatically detected terminal skin points of each perforator.*

*Secondary Evaluation, Page 7,*

*Because DSC and JSC values did not meet normality assumptions, non-parametric tests were used for all group comparisons, and Mann–Whitney U or Kruskal–Wallis tests were selected accordingly.*

The Results should specify whether segmentation performance differed between left and right thighs or across perforator calibers.

>> We thank the reviewer for this helpful comment. We have clarified the difference in segmentation performance between left and right thighs in the Results section, including statistical comparisons (p = 0.005 for DSC and p = 0.026 for JSC), as summarized in Table 3. Performance differences according to gender, perforator progression type, and BMI were also explicitly reported.

*Results, Page 8,*

*When analyzing the DSC and JSC values according to the left and right sides of the thigh, the DSC values were 69.73 ± 1.64 for the left side and 67.82 ± 1.58 for the right side (p = 0.005) and the JSC values were 68.95 ± 1.69 for the left side and 67.14 ± 1.89 for the right side (p = 0.026), which showed a higher degree of accuracy with the automatic segmentation on the left side. On the other hands, there were no statistical differences in DSC and JSC values according to gender, perforator progression type, and BMI (Table 3).*

*Discussion, Page 13,*

*Direct evaluation across perforator calibers was not feasible because the spatial resolution of CTA is insufficient to reliably measure diameter differences in distal perforator branches. This limitation should be addressed in future work using higher-resolution imaging or intraoperative validation.*

Figures could be improved by including visual overlays of automatic versus manual segmentations for representative cases.

>> We thank the reviewer for the valuable suggestion. To improve clarity, we revised the caption of Figure 1 to clearly describe the visual comparison between manual (red) and automatic (blue) detections presented in the representative CTA slices. The image resolution was also increased to 600 dpi to enhance visualization quality.

*Figure 1. Representative axial CTA slices showing the original image (left), manual perforator annotations (middle, red), and automatic detections generated by the deep learning model (right, blue). Manual and automatic localization showed close correspondence, although partial discontinuity was observed in distal arterial segments where vessel diameter becomes progressively smaller.*
